# *In Vivo* Force Application Reveals a Fast Tissue Softening and External Friction Increase during Early Embryogenesis

**DOI:** 10.1016/j.cub.2019.04.010

**Published:** 2019-05-06

**Authors:** Arturo D’Angelo, Kai Dierkes, Carlo Carolis, Guillaume Salbreux, Jérôme Solon

**Affiliations:** 1 Centre for Genomic Regulation (CRG), The Barcelona Institute of Science and Technology, Dr. Aiguader 88, Barcelona 08003, Spain; 2 Universitat Pompeu Fabra (UPF), Barcelona, Spain; 3 The Francis Crick Institute, 1 Midland Road, London NW1 1AT, UK

**Keywords:** force manipulation, magnetic tweezer, *Drosophila* embryo, cellularization, tissue mechanics, morphogenesis, cytoskeleton, vitelline envelope, continuum mechanics, physical modeling

## Abstract

During development, cell-generated forces induce tissue-scale deformations to shape the organism [1, 2]. The pattern and extent of these deformations depend not solely on the temporal and spatial profile of the generated force fields but also on the mechanical properties of the tissues that the forces act on. It is thus conceivable that, much like the cell-generated forces, the mechanical properties of tissues are modulated during development in order to drive morphogenesis toward specific developmental endpoints. Although many approaches have recently emerged to assess effective mechanical parameters of tissues [3, 4, 5, 6, 7, 8], they could not quantitatively relate spatially localized force induction to tissue-scale deformations *in vivo*. Here, we present a method that overcomes this limitation. Our approach is based on the application of controlled forces on a single microparticle embedded in an individual cell of an embryo. Combining measurements of bead displacement with the analysis of induced deformation fields in a continuum mechanics framework, we quantify material properties of the tissue and follow their changes over time. In particular, we uncover a rapid change in tissue response occurring during *Drosophila* cellularization, resulting from a softening of the blastoderm and an increase of external friction. We find that the microtubule cytoskeleton is a major contributor to epithelial mechanics at this stage. We identify developmentally controlled modulations in perivitelline spacing that can account for the changes in friction. Overall, our method allows for the measurement of key mechanical parameters governing tissue-scale deformations and flows occurring during morphogenesis.

## Highlights

• A method allows local force application and assessment of tissue mechanics *in vivo*
• A rapid blastoderm softening and increase in friction occur during cellularization
• Changes in friction correlate with modulations of perivitelline spacing

## Teaser

Assessment of tissue mechanical properties *in vivo* is challenging. D’Angelo, Dierkes et al. present a method based on local force application and on the analysis of induced deformations to measure epithelial mechanics. They find a rapid softening of the blastoderm and increase in external friction during the process of *Drosophila* cellularization.

## Results and Discussion

**Figure 1:**
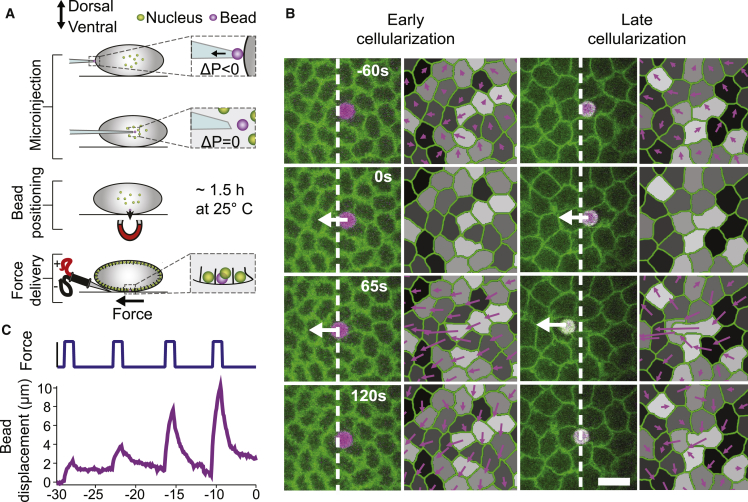
Force Applications on a Single Microparticle Induce Different Epithelial Responses (A) Injection procedure: an individual magnetic bead (purple) of 4.5μm diameter is injected into the yolk of an embryo at developmental stage 2. In order to position the bead apically, the embryo is let develop on top of a permanent magnet post injection (∼1.5 h at 25°C). When cellularization begins, force steps of 65 s duration are applied to the bead with an electromagnet. (B) Time-lapse images showing bead displacement and tissue deformation (purple arrows) in response to a force step (∼115 pN; onset at 0 s). Here, the bead was embedded into an individual cell of a Resille-GFP embryo. Force applications are shown for early cellularization (left; >16 min before gastrulation) and late cellularization (right; <16 min before gastrulation). White arrows indicate force application. White dashed lines mark the left side of the bead at time 0 s. Deformations are calculated relative to the cellular arrangement at the onset of force application (t = 0 s). Origins of the deformations are positioned on the cell center of mass. Scale bar, 10 μm. (C) Bead displacement for four consecutive force applications at ∼115 pN performed over the time course of cellularization (bottom) and corresponding force curve over time (top). Time is relative to the onset of gastrulation at t = 0 min. See also Figure S1, STAR Methods, and Videos S1 and S2.

To probe epithelial mechanics at early developmental stages, we have developed a protocol for injecting an individual magnetic microparticle into a single cell within a specific tissue of a living *Drosophila* embryo (Figures 1A, 1B, and S1G; Video S1; STAR Methods). After calibration (see STAR Methods and Figures S1A and S1B), we applied a controlled force step of 65-s duration and amplitude of about 115 pN to the magnetic bead by means of an electromagnet (Figure 1C; STAR Methods). Because the bead is coated, it can attach to the apical plasma membrane, and the force exerted on the bead is translated into a displacement parallel to the coverslip; uncoated beads are unable to stay apically (Figures S1C and S1D; STAR Methods). We obtained two complementary readouts characterizing the mechanical response of the tissue: (1) the bead displacement over time, and (2) the deformation field of the apical surface area of the epithelium (Figures 1B and 1C; STAR Methods).

Video S1. *Drosophila* Cellularization and *Drosophila* Larvae with a 4.5-μm Bead, Related to Figure 1 (Left) Combined XZ(top) and XY(bottom) view of a Resille GFP embryo undergoing cellularization after being injected with a 4.5 μm bead. Scale bar: 10 μm (Right) Transmission video of a *Drosophila* larvae pre-injected at pre-blastoderm stage. The white arrow indicates the bead position.

Applying forces at consecutive intervals in the blastoderm, spanning a period of 50 min before gastrulation in Resille-GFP-expressing embryos, we observe significant changes in both the displacement of the bead and the induced deformation field (Figures 1B and 1C; Video S2). Defining the origin of time as the onset of gastrulation, we find that the amplitude of bead displacement, i.e., the maximal displacement of the bead at the end of the force step relative to its position before the force step, changes abruptly from approximately 2μm to 8μm at around t = −16 min (Figures 1C and S2A). This change in maximal bead displacement is associated with a concurrent change in the spatial profile and range of the deformation and velocity fields (Figures 1B and S1E; Video S2). Our data therefore show that the same localized force can lead to a significantly different deformation pattern when applied at developmental time points that differ by only a few minutes.

**Figure 2:**
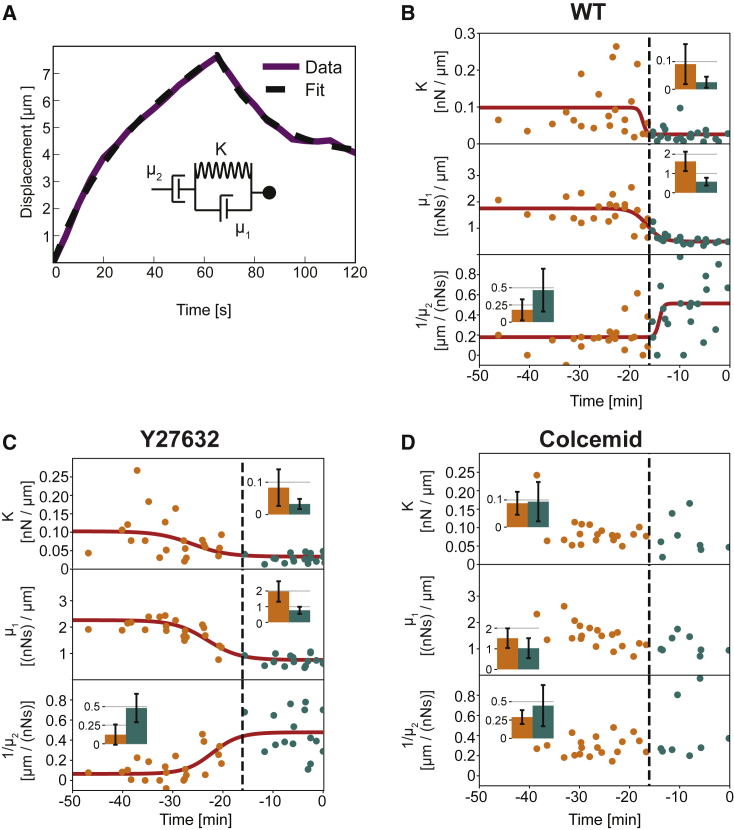
Effective Spring-Dashpot Model Reveals a Microtubule-Dependent Switch in Tissue Mechanical Properties during Cellularization (A) Representative example of a bead displacement curve after the application of force (∼115 pN; purple line) fitted with a Maxwell-Kelvin-Voigt model described by the three effective parameters $\mu_{1}$(0.61 nN.s/μm), $\mu_{2}$(1.99 nN.s/μm), and K (0.03 nN/μm) (see inset for a schematic of the corresponding rheological model). (B–D) Effective parameters as a function of developmental time relative to the onset of gastrulation. Each dot represents a single force application of ∼115 pN. In WT (B) and Y-27632 (C), we observe a step-like change of the three parameters at the onset of the fast phase of cellularization, i.e., around −16 min for WT and slightly before for Y-27632. This delay might be a consequence of time alignment: embryos injected with Y-27632 indeed display impaired and possibly delayed gastrulation, and therefore a shift of the chosen origin of time. Red lines are fits of experimental data to a sigmoid function. Black dashed lines represent the mean of the time point at the midstep of the three sigmoid curves for WT, which we use to distinguish between force application carried out in early (orange) and late (blue) phases of cellularization. The insets show average parameters over the early and late phases, with error bars indicating SDs. For Colcemid-treated embryos (D), there is no step-like change in mechanics between early and late cellularization, and a sigmoid fit of the data points could not converge. (WT_early_, 22 force applications on 8 embryos; WT_late_, 22 force applications on 11 embryos; Y-27632_early_, 24 force applications on 7 embryos; Y-27632_late_, 22 force applications on 7 embryos; Colcemid_early_, 19 force applications on 5 embryos; Colcemid_late_, 9 force applications on 3 embryos.) See also Figures S1 and S2, STAR Methods, and Videos S2 and S3.

**Figure 3:**
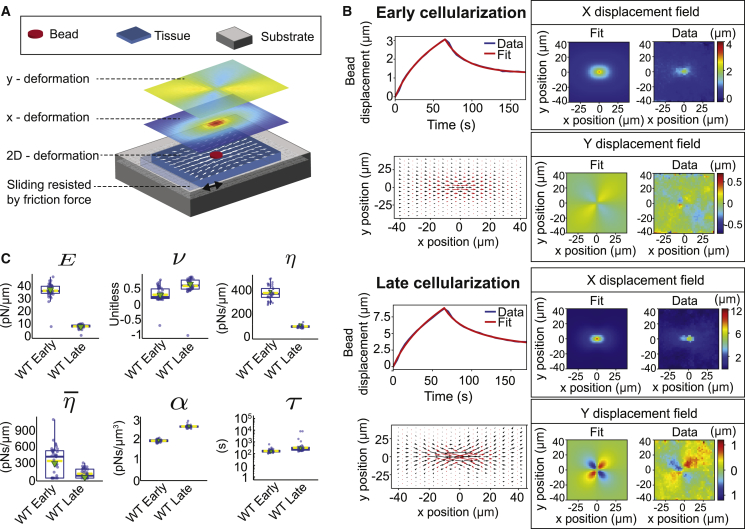
Analysis of the Tissue Deformation Field with a 2D Continuum Mechanics Framework Reveals a Softening of the Blastoderm and an Increase in External Friction (A) Schematic of the continuum description used to calculate deformation fields. The epithelial tissue is considered to be a 2D sheet moving relative to a substrate. Sliding of the tissue is resisted by friction. Bead-induced tissue deformation (represented by arrows) is decomposed into x and y deformations (shown as heatmaps). Mechanical tissue parameters are extracted from the experimental data by fitting the continuum description to both the averaged time evolution of bead displacement and the deformation field at the end of the force step. (B) Comparison of the experimental and continuum description of bead displacement and deformation fields at early (top) and late cellularization (bottom) in WT embryos. For both conditions, we display the average experimental and fitted bead displacement (top left), the fitted and experimental deformation field (bottom left; red and black arrows, respectively), and the fitted and average experimental x and y deformations (middle and right, top and bottom, respectively). (WT_early_, 10 force applications on 4 embryos and WT_late_, 10 on 5 embryos for the bead displacement; WT_early_, 10 force applications on 4 embryos and WT_late_, 8 on 5 embryos for the deformation fields.) (C) Mechanical parameters as extracted from the fit of our continuum description to experimental data. Values of the elasticity E, the Poisson ratio ν, the shear viscosity η, the bulk viscosity $\bar{\eta }$, the friction coefficient α, and the Maxwell viscoelastic timescale τ are shown for WT at early and late cellularization. Each blue dot is a single-fit value arising from the fit uncertainty analysis (STAR Methods). The boxplot encloses 50% of the data around the median, the yellow lines represent the mean of the data, and the green triangle shows the value obtained from the fit of the data. The upper/lower whisker extends from the hinge to the largest/smallest value no farther than 1.5 ^∗^ IQR from the hinge, respectively (where IQR is the inter-quartile range, or distance between the first and third quartiles). See also Figure S3, Videos S2 and S3, and STAR Methods.

Video S2. Force Application Experiment during WT *Drosophila* Cellularization, Related to Figures 1, 2, and 3 Time-lapse video showing a force application experiment on a Resille-GFP embryo at early cellularization (Left) and at late cellularization (Right). The purple arrow indicates when a force of ∼115 pN is applied on the bead. Scale bar: 10 μm.

To obtain a mechanical description of the response of the tissue upon force application, we employed a viscoelastic Maxwell-Kelvin-Voigt model for fitting the individual bead displacement curves (Figure 2A). In this mechanical circuit, a viscous element, with viscosity coefficient $\mu_{1}$, acts in parallel with an elastic element, with stiffness coefficient K, both operating in series with a viscous element described by a second viscosity coefficient,$\;\mu_{2}$ (Figure 2A; STAR Methods).

We determined the effective parameters introduced above for n = 44 force application experiments, performed at successive time points during cellularization in the ventral blastoderm, the process during which cellular membranes extend basally toward the interior part of the embryo [9, 10, 11]. Plotting the effective parameters as a function of developmental time reveals a rapid step-like change of all three mechanical parameters, occurring at about t = −16 min relative to the onset of gastrulation (Figure 2B). Indeed, we found that the observed time courses could be well fitted by sigmoids (Figure 2B). We verified that the observed mechanical switch and the value of the obtained parameters are largely independent of both the force amplitude and the number of force repetitions (Figures S1H, S2B, S2C, and S2I). We also noted that the effective viscoelastic timescales, $\mu_{1}/K$ and $\mu_{2}/K$, did not change significantly (Figure S2D). This observation indicates that both elasticity and viscosities change to the same extent, suggesting that these two parameters are not independent of each other. The observed switch in mechanical parameters coincides with a change in the velocity of the progression of cellularization [10] (Figures S1F and S2E). We therefore refer to the phases before and after the change in mechanical parameters as early and late cellularization.

To identify mechanisms underlying the origin of the switch in mechanical parameters, we decided to affect the cytoskeleton and the cellularization kinetics with pharmacological treatments. First, we affected the actin cytoskeleton with Latrunculin A. We observed a disruption of the blastoderm in the region close to the injection site; we therefore did not perform pulling experiments in this strongly perturbed condition (Figure S2G). We then attempted to affect actomyosin contractility using the rho-kinase inhibitor Y-27632. Upon treatment, we observed a delocalization of myosin from the apical site (Figure S2H) and a reduction in speed, compared to wild-type (WT) embryos, of the basal progression of the membrane occurring during cellularization (Figure S2E). Applying several force steps over developmental time, we observed a similar change, i.e., in bead displacement upon force application and mechanical parameters, to the changes observed in WT (Figure 2C; Video S3).

Video S3. Force Application Experiment during *Drosophila* Cellularization in Embryos Treated with Y-27632 and Colcemid, Related to Figures 2 and 3 Time-lapse video showing a force application experiment on a Resille-GFP embryo treated with Y-27632 and Colcemid. The purple arrow indicates force of ∼115 pN is applied. Scale bar: 10 μm

In the case of Colcemid-treated embryos, similarly to Royou et al. [9], we observed an absence of apicobasal membrane elongation during cellularization (Figure S2E). With our force experiments, we also observed an absence of noticeable change over time of the mechanical parameters estimated by the spring-dashpot model (Figure 2D). This shows that microtubules are essential for this switch to occur, and points toward the switch being associated with the process of cellularization.

To confirm that the switch in mechanical parameters is associated with membrane ingression during cellularization, we decided to impair cellularization through an alternative means, and injected an RNAi for slam, a known regulator of cellularization [10, 12]. In the slam RNAi-injected embryos, we observed defects in the process of cellularization (Figure S2E) and, with our mechanical probing methodology, we measured a shift in mechanical parameters between early and late cellularization that is less pronounced (Figure S2F).

Altogether, these results indicate that changes in mechanical properties are associated with furrow ingression occurring during cellularization and reveal that these changes are dependent on microtubules, rather than on actomyosin contractility.

The analysis of observed bead responses, using an effective rheological scheme, allows for the detection of rapid changes in the mechanical environment the bead is embedded in. However, effective response parameters are not direct readouts of actual material properties of the tissue, and do not allow the spatial deformation of the tissue to be related to the applied force. To overcome this limitation, we analyzed two complementary measurements of the tissue response in pulling experiments (bead displacement and tissue deformation) in the framework of a continuum description of tissue mechanics (Figure 3A; STAR Methods). Continuum mechanics descriptions have been successfully used to investigate epithelial deformations during morphogenesis [13, 14, 15]. In our description, the tissue is represented as a 2D viscoelastic sheet moving over a substrate with friction and the bead as a disk in the center of the 2D sheet.

We solved the dynamic equations of our model (Equations 7 and 8 in STAR Methods) and used a fitting procedure to adjust both the theoretically predicted bead displacement as a function of time and the displacement field at the end of force application to experimental results (Figure 3B; STAR Methods). We found that both the bead displacement and tissue deformation field could be reproduced by our continuum description for experiments performed in the early and late phases of cellularization and following pharmacological perturbations (Figures 3B and S3A).

We then compared the values of mechanical parameters characterizing the tissue at early and late stages (Figures 3C and S3B). In the WT case, we found a decrease in the elastic Young modulus (from E=36.8±6.1pN/μm to 7.0±1.7pN/μm) and in the shear viscosity (from η=383±60pN.s/μm to 96±11pN.s/μm), whereas the Maxwell viscoelastic timescale, the Poisson ratio, and the bulk viscosity remain comparable within the fit uncertainty (from τ=156±84 s to τ=293±1739s, from ν=0.28±0.23to ν=0.63±0.3, and from $\bar{\eta }=274\;\pm 262\text{ pN}.\text{s}/\text{μm}$ to 14 ±86 pN.s/μm; Figure 3C; STAR Methods). These changes are in line with our results from the spring-dashpot model and correspond to an overall softening of the epithelium, whereas the characteristic timescales of viscous to elastic transition ηΕ∼10s and long-time plasticity $\left(\tau ∼100\;\text{s}\right)$ do not vary significantly. In addition, we found that the friction coefficient increased between early and late cellularization in the WT case (from $\alpha =1.94\;\pm 0.07\;\text{pN}.\text{s}/{\text{μm}}^{3}$ to $\alpha =2.67\pm 0.08\;\text{pN}.\text{s}/{\text{μm}}^{3}$). As a result, the long timescale hydrodynamic length $\sqrt{E\tau /\alpha }$ that characterizes the spatial range of mechanically induced deformations in the tissue [16, 17] decreases from ∼54 μm to ∼28 μm over the course of cellularization. This indicates that, during early cellularization, a locally applied force induces deformations that propagate over more than 50 μm, representing typically 5 or 6 cell rows. In contrast, during late cellularization, a locally applied force induces deformations on a shorter-length scale, i.e., within 30 μm or typically 2 or 3 cell rows. These differences are readily visible in the range of deformation following force application on the bead (Figure 3B). Altogether, our method allows us to identify a tissue softening and an increase in external friction occurring during cellularization, which both contribute to reducing the characteristic range of tissue deformations.

We then applied our method to extract tissue mechanical parameters following Colcemid and Y-27632 treatments (Figure S3A). We find changes in tissue elastic moduli and viscosities that are generally in line with results obtained from the analysis of bead motion using the spring-dashpot model: tissue elastic and viscous moduli are unchanged following Y-27632 treatments, and tissue elasticity parameters obtained following Colcemid treatment resemble WT values at early stages. However, in Colcemid-treated embryos, we observed a decrease in shear viscosity compared to WT at early cellularization (Figure S3C; STAR Methods). This could be due to the suppression of the interactions between microtubule asters arising from different blastoderm cells at that stage [18]. Altogether, our method confirmed a significant tissue softening between early and late phases that is dependent on microtubules but not on actomyosin contractility. In addition, we noted that all treatments surprisingly modified the amplitude of the friction coefficient α (Figure S3C).

We wondered why the friction coefficient was changing over the course of cellularization and upon our pharmacological treatments, given that the friction coefficient reflects resistance to motion between the tissue and surrounding structures. The blastoderm is directly surrounded by the vitelline envelope, and therefore changes in contacts between the blastoderm and vitelline envelope could occur during cellularization. To estimate the width of the perivitelline space, we compared the position of the Resille-GFP signal with the position of the signal emitted by dextran Texas red injected into the perivitelline space (STAR Methods). In WT, we found that the peaks of the two signals move closer to one another during late cellularization, indicating a reduction in the space between the apical cell surface and the vitelline envelope, potentially increasing mechanical interactions and thus friction (Figures S4A and S4C).

To further test this hypothesis, we considered changes in friction that are visible following cytoskeletal perturbations: in Colcemid-treated embryos, the friction coefficient is increased, compared to WT values, at both early and late stages (with values $\alpha =6.16\;\pm 0.16\;\text{pN}.\text{s}/{\text{μm}}^{3}$and $\alpha =5.0\;\pm 0.19\text{ pN}.\text{s}/{\text{μm}}^{3}$, for the early and late stages, respectively), and in Y-27632-treated embryos, the friction coefficient is increased compared to WT at the early stage $\left(\alpha =3.42\;\pm 0.2\;\text{pN}.s/{\text{μm}}^{3}\right)$ but decreased at the late stage $\left(\alpha =1.89\;\pm 0.04\;\text{pN}.\text{s}/{\text{μm}}^{3}\right)$ (Figure S3C). We then tested whether these changes were consistent with changes in the width of the perivitelline space in Colcemid- and Y-27632-treated embryos. In the case of Colcemid, we measured a shorter distance between the blastoderm and the vitelline membrane at early cellularization compared with WT, consistent with a higher friction value (Figures S4A and S4C). For Y-27632, we observed temporal changes that were opposite the WT: we measured smaller distances at the early stage and larger distances at the late stage, again consistent with a higher friction coefficient at early cellularization and a lower friction coefficient at late cellularization (Figures S4A and S4C). Overall, we observe an inverse correlation between measured friction coefficients and perivitelline space widths (Figure S4B), suggesting that cytoskeletal components can affect the distance between the tissue and the vitelline membrane, and consequently external friction.

How could such changes in vitelline space occur during cellularization? During this process, microvilli present at the apical surface of the cells are removed, leading to a flattening of the apical cell surface [10, 19]. This change in cellular surface architecture could influence the strength of the interaction between the blastoderm and the vitelline envelope surrounding it. We thus decided to perform high-resolution transverse imaging of the apical surface of the blastoderm in WT at early and late cellularization in Colcemid- and Y-27632-treated embryos using GAP43-mCherry as a membrane marker.

**Figure 4:**
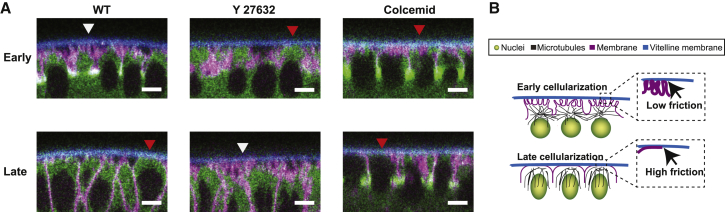
Apical Cell-Surface Morphology of the Blastoderm Cells in WT, Y-27632, and Colcemid Treated Embryos (A) XZ snapshots of the ventral blastoderm at early and late cellularization showing GAP43-mCherry (in magenta), SqhGFP (in green), and the autofluorescence of the vitelline membrane (in blue) for WT and Y-27632 and Colcemid treated embryos. White arrowheads highlight regions with microvilli, showing high membrane ruffles and limited contact zone with the vitelline membrane. Red arrowheads highlight regions without microvilli, where the contact zone of the blastoderm apical cell surface with the vitelline envelope is enlarged. Scale bar, 5 μm. (B) The sketch represents a simplified cross-sectional view of the WT embryo at early and late cellularization. At early cellularization, microvilli are present showing high membrane ruffles at the apical blastoderm, there is a larger distance between the tissue and the vitelline membrane (as seen in Figures S4A and S4C), and a small friction coefficient α is measured (see Figure 3C). In contrast, in late cellularization, microvilli and associated membrane ruffles are removed from the apical blastoderm, resulting in a larger contact zone between cells and the vitelline envelope, the average distance from the vitelline membrane is reduced (Figures S4A and S4C), and a high friction coefficient α is measured (Figure 3C). See also Figure S4 and Video S4.

Consistent with previous studies [10, 19], during WT early cellularization, we observed the presence of microvilli at the apical surface and found that they form a “cushion” between the apical surface and the vitelline envelope (Video S4). At late cellularization, in contrast, the microvilli are not present anymore and the apical membrane of the cell appears flat and in close contact with the vitelline envelope (Figure 4A; Video S4). When treated with Colcemid, the apical cell surface does present altered microvilli at early cellularization and appear flat, in close contact with the vitelline envelope, similar to WT blastoderm cells at late cellularization (Figure 4A; Video S4). We also observed modified nuclei positions in Colcemid-treated embryos, with nuclei lying closer to the vitelline envelope, potentially increasing the cell-vitelline envelope interaction (Figure 4A). When treated with Y-27632 inhibitor, blastoderm cells also present altered microvilli at early cellularization and apparent close contacts between the cells and the vitelline envelope. In contrast with WT, a cushion between the apical surface and the vitelline envelope is not removed at late cellularization but rather increases with microvilli apparent at the apical surface, possibly acting as spacers (Figure 4A; Video S4).

Video S4. Transverse Video of *Drosophila* Cellularization in WT and Y-27632- and Colcemid-Treated Embryos, Related to Figure 4 XZ video of the ventral blastoderm during cellularization labeled with GAP43-mCherry (in magenta), Sqh-GFP (in green) and showing autoflorescence from the vitelline membrane (in blue). Time 0 indicates the beginning of gastrulation. Time interval: 2 min, scale bar: 5 μm

Overall, we observe that when a low friction coefficient is measured (in WT early cellularization and Y-27632 late cellularization), a cushion of microvilli is present apically and the blastoderm-vitelline distance is large. On the other hand, when a high friction coefficient is measured (in WT late cellularization, Y-27632 early cellularization, and Colcemid early and late cellularization), the microvilli are altered or absent and the vitelline-blastoderm distance is reduced.

Altogether, our results indicate that the changes in the external friction coefficient observed during cellularization could arise from changes in the blastoderm-vitelline envelope interaction (Figure 4B). More specifically, we identified the actomyosin and microtubule cytoskeletons as essential components for the control of the architecture of the apical cell surface of the blastoderm and the perivitelline spacing.

Here, we have presented a methodology for applying controlled forces, extracting tissue-scale deformations, and measuring mechanical properties of epithelia within a continuum mechanics framework. Our method allows for the *in vivo* measurement of essential physical parameters governing tissue-scale deformations and flows, and therefore relates force patterns to spatial deformation profiles. By monitoring mechanical changes during development, we uncovered a rapid step-like softening of the blastoderm preceding the onset of gastrulation. Our work therefore indicates that, in addition to the modulation of cellular forces, mechanical properties of tissues can change significantly during morphogenesis, on timescales on the order of minutes. Such changes must impact morphogenetic processes, as the pattern and amplitude of deformations in response to a given force field are set by the mechanical properties of the involved tissues. Indeed, low tissue stiffness and high external friction reduce the range of propagation of deformations, and therefore allow for local deformations that do not impact cells at larger distances. This feature could be important to restrict the propagation of deformations when several morphogenetic rearrangements occur concurrently, such as during gastrulation. In addition, our results identify a cellular control of the mechanical interaction with surrounding structures such as the vitelline envelope. Evidence of interaction between epithelia and surrounding structures such as the extracellular matrix (ECM) has previously been identified in the context of wing disc morphogenesis [20, 21]. The mechanical interaction of tissues with surrounding material, and therefore the role of frictional forces, appears an essential component of morphogenesis [22]. Our work allows the assessment of these frictional forces and shows that friction could be actively modulated during development by cellular processes. We propose that such modulations in frictional forces, by changing the force balance with other forces acting during development, could directly impact morphogenesis.

Mechanical properties of tissues have been shown to be strongly influenced by the actin cytoskeleton [23, 24]. Our data indicate that the actin cytoskeleton is essential for the maintenance of blastoderm stability. Remarkably, in contrast with other epithelia, our results obtained from our 2D analysis of drug-injected embryos identified the microtubule network as one of the factors determining tissue mechanics at this stage.

Our work therefore suggests that some of the observed modulation of tissue mechanics could be due to the rearrangement of the microtubule network occurring during cellularization [25, 26]. Microtubules emanating from the asters associated with different nuclei display transient connections that disappear during late cellularization [18]. A highly connected microtubule meshwork at early cellularization thus rearranges into a network of only weakly interacting microtubule asters. One hypothesis is that this change in connectivity is one of the causes of changes in mechanical properties. In addition, the microtubule cytoskeleton reorganization is essential for furrow ingression and basal migration of the actin meshwork. Our data suggest that the basal displacement of the actin cytoskeleton could be at the origin of the observed switch in tissue mechanical properties at the apical site of the blastoderm.

Finally, our method for mechanical measurements, due to its versatility, low cost, and adaptability to different microscopy techniques, can easily be employed in other systems. It thus paves the way for further studies mapping out epithelial mechanics.

## STAR★Methods

### Key Resources Table

**Table:** 

REAGENT or RESOURCE	SOURCE	IDENTIFIER
**Antibodies**		

GBP GFP binding protein	This study	N/A

**Chemicals, Peptides, and Recombinant Proteins**		

Colcemid	Santa Cruz Biotechnoloy	Cat#sc-202550
Y-27632	Sigma Aldrich	Cat# Y0503-5MG
Latruculin	Thermo Fisher Scientific	Cat# L12370
SLAM RNAi	This study	N/A
Dextran Texas Red 70000MW	Thermo Fisher Scientific	D1864

**Critical Commercial Assays**		

Dynabeads M450 tosylactivated	Thermo Fisher Scientific	Cat#14013

**Experimental Models: *Drosophila melanogaster***		

Resille GFP	Marta Llimargas Lab [27]	N/A
Mat GAL4 Sqh GFP: Mat GAL4 GAP mcherry	Adam Martin Lab [28]	N/A
Sqh^Ax3^, Sqh-GFP	Damian Brunner Lab [9]	N/A

**Software and Algorithms**		

Fiji	Fiji	https://fiji.sc
Python	Python	https://www.python.org/
Packing analyzer V2.0	Suzanne Eaton Lab [29]	N/A
R	R	https://www.r-project.org/
MATLAB	MATLAB	https://www.mathworks.com

### Contact for Reagent and Resource Sharing

Further information and requests for resources and reagents should be directed to and will be fulfilled by the Lead Contact, Jérôme Solon (jerome.solon@crg.es)

### Experimental Model and Subject Details

*Drosophila melanogaster* embryos were used in this study. All analyzed embryos were at early embryonic stage (stage 5 of embryonic development).

Health/immune status: not applicable.

Subjects were never involved in previous procedures.

All *Drosophila* flies were drug and test naive.

The genotypes of the strains used in this study are detailed in the section below.

Husbandry/housing conditions: *Drosophila* strains were maintained at 18°C in the laboratory *Drosophila* stocks and at 25°C in preparation of the experiments. Experiments were performed at room temperature (22°C).

#### Fly Strains

The following lines were used: Resille-GFP [27] also known as P*{PTT-un1}jCG8668* ^*117∼2*^, P(w+sqh::GFP)42,mat67;P(Gap43::mCherry)mat15 [28] and Sqh^Ax3^, Sqh-GFP [9].

#### Preparation of *Drosophila* Embryos

To inject the bead at pre-blastoderm stage, the embryos are collected during 15min, dechorionated in 100% bleach and mounted on an heptane/glue coverslip [30]. The tissue in which the bead will be located has to face the coverslip. Once mounted, the embryos are dehydrated for 10 min at 25°C and then covered with Voltalef 10S oil.

### Method Details

#### Overview

All experiments were replicated at least three times. Statistics are detailed in the respective figure legends. No strategy was used for randomization or stratification; no blinding was used, and sample-size were not estimated. As a criterion for considered data, for pulling experiments, the magnetic bead has to be attached at the apical cell membrane. Experiments where the magnetic bead detached from the cell membrane, attached on a wrong cell membrane position for pulling or when the bead displaced along the apico-basal axis were excluded. Deformation fields showing a drift independent of the pulling force were also excluded. For Figure S2D, five outlier points with extreme values $\left(∼10^{9}\;\text{s}\right)$ have been excluded from the graph.

#### Particle microinjection

Micro-needles are generated by pulling 1mm glass capillaries (Narishige G1) using a micro-puller (Sutter instruments P30). The needle tip is then opened and beveled in a controlled manner to facilitate the injection using a micro grinder (Narishige EG-44). The internal diameter is set to be slightly smaller than the particle, i.e., 3.5-4 μm (Figure 1A). This allows us, by tuning the pressure inside the micro-needle with a microinjector (WPI PV 820), to hold and inject an individual particle within the *Drosophila* embryo. Once inside the embryo, the bead can be oriented on the A-P and dorso-lateral axes using an electromagnet. To specifically target intracellular compartments, the microparticle was coated with a GFP nanobody [31], which specifically binds GFP-tagged proteins. After injection, the embryos are positioned above a permanent magnet and are left to develop (2h to apply force during cellularization) in a wet chamber at 25°C. By choosing the orientation of the embryo relative to the permanent magnet, we can cause the bead to be encapsulated in preselected groups of cells that will develop into specific tissues at a later stage.

#### Calibration of the electro-magnet

To induce controlled forces we designed an electromagnet similarly to previous *in vitro* studies [32]. A core of soft metal with a tip shape (Mumetal, Sekels Gmbh) is surrounded by 100 coils of copper cable fed by a power supply in order to generate a solenoid. An additional radiator is placed between the soft metal core and the copper coils to evacuate the thermal dissipation. The electric current circulating within the copper coils will generate a magnetic field that will be focused at the tip of the soft metal core. The magnetic force exerted on the paramagnetic micro-particle is directly proportional to the gradient of the magnetic field [32]. Therefore, the force would be highest at the tip and decay as a power law with the distance from the tip of the electro-magnet (Figure S1B). In order to calibrate the magnetic force exerted on the micro-particle, we have established a calibration assay using micro-particles embedded in PDMS (Sigma Aldrich). Because its viscosity is well established, by measuring the velocity of the micro-particles within the magnetic field generated by the electro-magnet, one can determine precisely the force applied to the particle as a function of its distance from the tip for a specific current applied to the solenoid. For a current of 0.3 A, the typical force-distance curve ranges from ∼1 nN at 60 μm from the tip to ∼100 pN at 200 μm (Figures S1A and S1B). Due to geometrical constrains arising from the embryo shape, the typically used bead-magnet distances in our experiments were about 190 μm (Figure S1B, inset). This corresponds to forces of ∼115 pN (high force condition, for 0.3 A) and ∼50pN (low force condition, for 0.15 A). Note that for currents above 0.5 A the magnetic force saturates.

#### Imaging and force application

Embryos were imaged at room temperature (22°C) using an Andor spinning disk confocal microscope. Z stacks of 4-5 sections with a 1 micron spacing interval were collected every 5 s with 100X magnification.

The magnet positioning was controlled using a three-axis micromanipulator (Narishige UMM-3FC) mounted on the microscope stage. In these experiments, the electromagnet was positioned approximately at 190 μm from the bead, and the force was applied systematically for 65 s (Figure S1B). Experiments were only considered valid for analysis when the bead was seen to be attached to the apical cell membrane. On some occasions the bead detached from the membrane and became displaced in the basal direction or became attached to an inappropriate site in the cell for the pulling experiment. In these cases, we did not include the data in our analysis. Specifically, to avoid potential artifacts resulting from tether formation, we only considered experiments in which the bead was pushing on the lateral plasma membrane and excluded experiments in which the bead was pulling on trailing plasma membrane.

Imaging of the perivitelline space with Dextran Texas-Red 70000MW (Molecular Probes) and Resille-GFP was performed on an Andor spinning disk confocal microscope (with a Z resolution of 0.5μm and a magnification of 63X).

XZ high-resolution transverse timelapse sequences were taken on a Leica TCS SP8 confocal microscope (stand: DMI-8) with a 93x 1.3 NA glycerol immersion objective with motorized correction collar for compensation of refractive index mismatches. The channels were acquired in line-by-line sequential imaging mode using a pulsed white light laser with 560 nm and 480 nm wavelengths. Cell membranes were imaged with the mcherry channel, myosin levels with the GFP channel and vitelline envelope using the autofluorescence signal emanating at 600nm when excited at 480nm. Fast XZ scanning was done with galvanometric z-stage of the SP8 confocal over a z- range of ∼20 μm. Images were taken at 2 minute intervals.

#### Drug Microinjection

Developing embryos were injected in the early phase of cellularization using a Femtojet Injector (Eppendorf). Injected concentrations were as follows: Rho-K inhibitor (Y-27632 Sigma Aldrich) has been injected at 30 mM, Colcemid (Santa Cruz Biotechnology) has been injected at 1 mM, SLAM RNAi has been injected at 500 μg/μL and Dextran Texas-Red 70000MW (Molecular Probes) has been injected at concentrations between 7 and 25 mg/ml. We estimate ∼20-fold dilution in the embryo. For Rho-K inhibitor cases, embryos were selected for phenotype showing impairment/absence of contraction at gastrulation.

### Quantification and Statistical Analysis

Statistical significance was calculated using a t test for pairwise comparison. Significance: NS = not significant, ^∗^p < 0.05, ^∗∗^p < 0.01, ^∗∗∗^p < 0.001, ^∗∗∗∗^p < 0.0001. Error bars are standard deviations, except for the boxplots.

#### Analysis of the bead displacement

Analysis of the bead displacement was performed using Fiji. Tracking of the bead was performed on images acquired from the red channel using the auto-fluorescence emission spectrum of the bead. The following analysis step were implemented: (1) A median filter (radius 2) was applied to maximum intensity projections of each z stacks; (2) the projection was then manually thresholded; (3) The MTrack2 plugin was used to automatically extract the x-y coordinate of the bead.

On some occasions, samples exhibited a background drift in the location of the tissue. In order to correct for this, a linear function, f(x)=mx+c, was fitted to the bead displacement in the 100 s preceding the force application. Assuming this drift persisted during the force application, we subtracted this linear trend from the measured bead displacement.

#### Fit of the changes in mechanical parameters

The fit of the changes in values of three mechanical parameters K, $\mu_{1}$and $\left(1/\mu_{2}\right)$ over time in Figures 2B and 2C was performed with the following sigmoid function:

$$f\left(t\right)=P_{0}\left(\frac{1}{1+P_{1}e^{-P_{2}t}}\right)+P_{3}$$

The time in the middle of the step is given by $\text{ln}\left(P_{1}\right)/P_{2}$. We defined the transition time from early to late phases as the average of the time in the middle of the step resulting from the fit of the three parameters.

#### Analysis of the experimental deformation field

Experimental images where aligned using the position of the bead at t = 0 s. Cell shapes were determined from the outlines of cell membranes at the onset and at the end of the period of force application, i.e., at t = 0 s and t = 65 s, respectively, using the software Packing Analyzer v.2.0 [29]. Cells were tracked using the same software. We determined the displacement vector, d(x), corresponding to the centroid movement between t = 0 s and t = 65 s of all cells within the field of view. Here, x=(x,y) is a vector denoting the position of the centroid of the cell at t = 0 s, relative to the bead position at t = 0 s. We considered the total set of obtained deformation vectors, $\left\{d\left(x_{k}\right)\;\text{for}\;k=1,\ldots ,M\right\}$, as the readout of the experimental deformation field. Note that cells have a typical diameter of about ∼5μm, which then defines the spatial resolution of the deformation field measurement.

#### Vitelline envelope-apical cell surface distance estimation analysis

The estimation of the distance between the vitellin envelope and apical cell surface was performed using a z-reslice along the AP axis (using FIJI) applied to z stacks with a spacing of 0.5 μm, taken on embryos expressing Resille-GFP and injected with Dextran Texas-Red 70000MW (Molecular Probes) in the perivitelline space. We use the fluorescence intensity of Dextran Texas Red as a proxy for the position of the perivitelline space. Using a custom made MATLAB script, an average z-fluorescence profile is obtained by: 1) performing a sliding average of 5 pixels along the z-reslice and 2) by realigning in z each local average to the maximal dextran-RFP fluorescence intensity. A spline interpolation was then performed on the Resille-GFP intensity curves to determine the position of the maximum of intensity, accounting for the shape of the fluorescence intensity peak, used as the location of the apical cell surface.

#### Spring and dashpot model

We obtain here the equation relating force and deformation in the spring and dashpot model indicated in Figure 2A. In such a rheological scheme, the behavior at long-time is a fluid relaxation, occurring on a timescale dictated by the ratio of the viscosity parameter $\mu_{2}$ to the stiffness coefficientK. Introducing a long-time viscous response was necessary to account for the incomplete relaxation of the bead after application of the force, which indicates that the tissue does not behave like a purely elastic material on long timescales.

The force F exerted on the system is related to its displacement U by

(Equation 1) $$\left(\frac{K}{\mu_{2}}+\frac{\mu_{1}+\mu_{2}}{\mu_{2}}\frac{d}{dt}\right)F=\left(K+\mu_{1}\frac{d}{dt}\right)\frac{dU}{dt}$$

We then solve this equation for a step function of force application:

(Equation 2) $$\begin{array}{c} F\left(t\right)=0\;\;\;\;\;t<0\;\;\;\;\;\;\;\;\;\;\;\;\;\;\;\;\;\;\;\;\\ =F_{0}\;\;\;\;\;0\leq t\leq T\\ =0\;\;\;\;\;t>T,\;\;\;\;\;\;\;\;\; \end{array}$$

where T is the time of force application. Taking the initial condition U(t)=0, we then find the following deformation:

(Equation 3) $$U\left(t\right)=\{\begin{array}{c} \frac{F_{0}}{\mu_{2}}\left(t+\frac{\mu_{2}}{K}\left(1-e^{-\frac{Kt}{\mu_{1}}}\right)\right)\;\;\;\;\;t<T\\ \frac{F_{0}}{\mu_{2}}\left(T-\frac{\mu_{2}}{K}e^{-\frac{Kt}{\mu_{1}}}\left(1-e^{\frac{KT}{\mu_{1}}}\right)\right)\;\;\;\;\;t>T \end{array}$$

This solution is fitted to experimental data of bead displacement as a function of time to obtain $\mu_{1}$, $\mu_{2}$, K, using the value of the pulling force $F_{0}$ measured before each pulling experiment session. The average values of the pulling forces for each condition are indicated in the table below.

**Table:** Table of average force values used for the fit and number of individual force applications.

Type	Stage	Average force	No. of applications	No. of embryos
WT low force	Early	43.26±5.25pN	7	3
WT low force	Late	43.13±5.52pN	10	4
WT high force	Early	114.5±5.11pN	22	8
WT high force	Late	111.15±8.9pN	22	11
Colcemid	Early	113.45±11.21pN	19	5
Colcemid	Late	125.97±9.7pN	9	3
Y-27632	Early	124.55±11.48pN	24	7
Y-27632	Late	132.85±17.52pN	22	6

Not that for purely elastic cases, $\mu_{2}$ would diverge toward infinity, we therefore plot the fluidity 1μ2 in Figures 2B, 2C, 2D, S2B, S2F, and S2I.

#### 2D Physical description of tissue deformation

In our description, we represent the tissue as a two-dimensional flat sheet (see Figure 3A). Below, we denote spatial coordinates x,y by latin indices and the associated cartesian basis by $e_{x},\;e_{y}$. The bead is represented by a disc with surface area S, exerting a homogeneous force per unit area $f_{j}=F_{j}/S$ on the tissue, with $F_{j}$ the total force exerted by the bead. In our model, the tissue moves relative to an external fixed substrate, representing the external medium, which exerts a dynamic friction force acting against motion of the tissue, with friction coefficient α. The displacement of the tissue is denoted by the vector **u**(*x,y*) and its velocity denoted by the vector **v**(*x, y*). To describe the stress distribution and response of the tissue, we consider the following constitutive equation for the two-dimensional tissue stress tensor $t_{ij}$:

(Equation 4) $$\left(1+\tau \frac{D}{Dt}\right)t_{ij}=\frac{E\tau }{1+\nu }\left[v_{ij}+\frac{\nu }{1-\nu }v_{kk}\delta_{ij}\right]+\tau \frac{D}{Dt}\left[2\eta \left(v_{ij}-\frac{1}{2}v_{kk}\delta_{ij}\right)+\bar{\eta }v_{kk}\delta_{ij}\right]\;,$$

where $v_{ij}=\left(\partial_{i}v_{j}+\partial_{j}v_{i}\right)/2$ is the symmetric velocity gradient tensor. In the equation above, (D/Dt) is the corotational derivative:

(Equation 5) $$\frac{D}{Dt}t_{ij}=\frac{\partial }{\partial t}t_{ij}+v_{k}\partial_{k}t_{ij}+\omega_{ik}t_{kj}+\omega_{jk}t_{ik}$$

In the following, we neglect for simplicity the non-linear advective and corotational terms, and relate the deformation field to the velocity field by $v=\partial_{t}u$.

Equation 4 reflects our assumptions on the rheological behavior of the tissue. We assume that on short timescales, the tissue has a viscous response with shear and bulk viscosities η and $\bar{\eta }$. The bulk viscosity describes the resistance to flows resulting in local changes in tissue area, and the shear viscosity the response to tissue flows with conserved area. On intermediate timescales, above a characteristic time ∼η/E, the tissue has an elastic response, with a deformability characterized by the two-dimensional Young’s modulus E and the two-dimensional Poisson ratio ν. On longer timescales above the Maxwell viscoelastic timescale τ, we assume that elastic stresses can relax, for instance due to cell rearrangements, allowing cell deformations to decrease.

The tissue is described here as a linear material, implying that its mechanical response scales with the amplitude of the force. To test whether our assumption of a linear mechanical response of the tissue holds, we quantified bead displacement and tissue deformations induced by lower force application (50pN instead of 115pN; Figure S2B). We found that the obtained deformation profiles at the two stages were roughly proportional to the force application on the bead, consistent with a linear mechanical response (Figure S2C).

In Equation 4, we consider a tissue rheology where each element of tissue has a rheology equivalent to the effective spring-dashpot model used in the previous section (Figure 2A). However, the continuum description takes into account the spatial distribution of stresses, and it distinguishes explicitly between dissipative processes internal to the tissue (characterized by viscosities) and external to the tissue (characterized by a friction coefficient). In addition, Equation 4 discriminates between isotropic and anisotropic elastic moduli (determined by E and ν) and isotropic and anisotropic viscosities (defined by the bulk and shear viscosities). Indeed, local changes in apical surface area of the tissue by isotropic compression/expansion do not necessarily result in the same resisting forces as similar changes via anisotropic elongation/contraction that maintain a constant surface area.

The force balance within the tissue reads

(Equation 6) $$\partial_{i}t_{ij}+f_{j}=\alpha v_{j}$$

where $f_{j}$ and $-\alpha v_{j}$ are external force densities acting on the tissue, and $\partial_{i}t_{ij}$ is the contribution of internal stresses to local force balance.

Combining these two equations, we get the dynamical equation for the velocity field

(Equation 7) $$\frac{E\tau }{2\left(1+\nu \right)}\left(\partial_{i}^{2}v_{j}+\frac{1+\nu }{1-\nu }\partial_{j}\partial_{i}v_{i}\right)+\left(1+\tau \frac{\partial }{\partial t}\right)f_{j}=\alpha \left(1+\tau \frac{\partial }{\partial t}\right)v_{j}-\eta \tau \partial_{i}^{2}\frac{\partial v_{j}}{\partial t}-\bar{\eta }\tau \partial_{j}\partial_{k}\frac{\partial v_{k}}{\partial t}$$

In the following, we consider the following external force density **f**(*t,x,y*) exerted by the bead:

(Equation 8) $$f\left(t,x,y\right)=\frac{F\left(t\right)}{\pi R^{2}}H\left(x,y\right)e_{x}$$

where H(x,y)=1 on a disk of radius R=2.25μm centered at (0,0), and is 0 elsewhere, and F(t) is the total force exerted by the bead, defined in Equation 2. We assume here for simplicity that the force acting on the bead results in a homogeneous two-dimensional force density acting on the tissue.

#### Numerical resolution

We solve for the deformation field introduced by the force density **f** in a two-dimensional square box of size *L*, with periodic boundary conditions. Introducing the Fourier transform of the velocity and force density field,

(Equation 9) $${\tilde{v}}_{i}\left(q\right)=\int dxv_{i}\left(x\right)e^{-iq\cdot x},\;\;\;{\tilde{f}}_{i}\left(q\right)=\int dxf_{i}\left(x\right)e^{-iq\cdot x},\;$$

the Fourier transformed system reads

(Equation 10) $$\left(\begin{array}{c} 1+l^{2}\left(q^{2}+\lambda q_{x}^{2}\right)\\ l^{2}\lambda q_{x}q_{y} \end{array}\begin{array}{c} l^{2}\lambda q_{x}q_{y}\\ 1+l^{2}\left(q^{2}+\lambda q_{y}^{2}\right) \end{array}\right)\frac{\partial }{\partial t}\left(\begin{array}{c} {\tilde{v}}_{x}\\ {\tilde{v}}_{y} \end{array}\right)=-\frac{1}{\tau }\left(\begin{array}{c} {\tilde{v}}_{x}\\ {\tilde{v}}_{y} \end{array}\right)-D\left(\begin{array}{c} q^{2}+\beta q_{x}^{2}\\ \beta q_{x}q_{y} \end{array}\begin{array}{c} \beta q_{x}q_{y}\\ q^{2}+\beta q_{y}^{2} \end{array}\right)\left(\begin{array}{c} {\tilde{v}}_{x}\\ {\tilde{v}}_{y} \end{array}\right)+\left(\frac{1}{\tau \alpha }+\frac{1}{\alpha }\frac{\partial }{\partial t}\right)\left(\begin{array}{c} {\tilde{f}}_{x}\\ {\tilde{f}}_{y} \end{array}\right)\;,$$

where we have introduced $\beta =\left(1+\nu )/(1-\nu \right)$, $D=E/(2\alpha \left(1+\nu \right))$, $l=\sqrt{\frac{\eta }{\alpha }}$, $\lambda =\bar{\eta }/\eta$, and we use the notation $q^{2}=q_{x}^{2}+q_{y}^{2}$. The Fourier transform of the force density distribution introduced in Equation 8 reads

(Equation 11) $${\tilde{f}}_{x}=\left\{ \begin{array}{c} F\left(t\right)\frac{2j_{1}\left(R\sqrt{q_{x}^{2}+q_{y}^{2}}\right)}{R\sqrt{q_{x}^{2}+q_{y}^{2}}}\;\;for\;\;q_{x}^{2}+q_{y}^{2}>0\\ \;\;\;\;\;\;\;\;\;\;\;\;\;\;\;F\left(t\right)\;\;\;\;\;\;\;\;\;\;\;\;\;\;\;\;\;\;\;for\;\;q_{x}^{2}+q_{y}^{2}=0 \end{array}\right.$$

(Equation 12) $${\tilde{f}}_{y}=0$$

where $j_{1}$ denotes the Bessel function of the first kind of order 1. The function F(t) is taken to be a function of time as in Equation 2. Note that as a result, a discontinuous jump of velocity occurs at *t* = 0. The value of the velocity at *t* = 0^+^ can be obtained by integrating Equation 10 in time between −ϵ and ϵ with ϵ>0, and letting ϵ→0. A similar approach can be used at t=T. We then solve Equation 10 for a given domain size *L* with a spatial resolution of Δx=1μm and Δy=1μm, using a forward Euler scheme with a time-step of Δt=0.01s. Applying an inverse Fourier transform, we then obtain a solution for the deformation field $u_{p}\left(t,x,y\right)$, obtained by integrating the velocity field, depending on the choice of parameter vector

p=(β,D,l,λ,α,τ).

#### Fitting Procedure

We experimentally measure the displacement of the bead as a function of time, denoted $\left(d\left(0\right),d\left(t_{1}\right),\ldots ,d\left(t_{N}\right)\right)$, and the displacement field in the tissue around the bead at T=65s, characterized by the set of displacement vectors $\left(d\left(x_{1}\right),\ldots ,d\left(x_{\text{M}}\right)\right)$, where $x_{k}$ is a set of measurements points in the tissue. We then define the objective function

(Equation 13) $$S\left(p\right)=\frac{1}{M}\sum_{k=1}^{M}{\left(d\left(x_{k}\right)-u_{p}\left(T,x_{k}\right)\right)}^{2}+\frac{1}{N}\sum_{k=0}^{N}{\left(d\left(t_{k}\right)-u_{x,p}\left(t_{k},0,0\right)\right)}^{2}.$$

In order to obtain fit values for the parameter vector p=(β,D,l,λ,α,τ), we use a custom code written in Python. Note that in our fit procedure, we use unitless parameters in order to avoid difficulties stemming from the fact that different parameters differ by orders of magnitude in their numerical value. For each dataset, we ran five independent fit procedures using the L-BFGS-B algorithm with different initial conditions. Initial values where drawn randomly within ± 75% of a fixed set of parameters that was chosen to give results comparable but different to the experimental data. We then chose the parameter vector that resulted in the lowest value of the objective function *S*. The average forces used for the fits are 112.6 pN for WT early, 106.3 pN for WT late, 110.8 pN for Colcemid early, 122.6 pN for Colcemid late, 125.8 pN for Y-27632 early and 111.5 pN for Y-27632 late. These values are obtained from average force application in experiments used for the fitting procedure described here, which are a subset of experiments used for the spring-dashpot fitting procedure (Table above). In order to test how fit parameters depend on the domain size *L*, we ran our minimization protocol for various choices of *L* (see Figure S3B). We found that parameter values converge for increasing values of *L* and are almost constant for *L* > 350 μm. The values reported correspond to fit results obtained at *L* = 400 μm. A comparison of experimental and fit bead displacement and deformation field yields a good agreement (see Figures 3B and S3A). The resulting parameters of the fit are indicated in the Table below. In the last line of the Table we indicate the value of the characteristic length $\sqrt{E\tau /\alpha }$ obtained from fit parameters.

**Table:** 

	WT, early	WT, late	Colcemid, early	Colcemid, late	Y27632, early	Y27632, late
E[pN/μm]	36.8, std = 6.1	7.0, std = 1.7	27.4, std = 4.4	22.0, std = 6.5	33.9, std = 10.6	10.7, std = 1.3
ν	0.28, std = 0.23	0.63, std = 0.30	0.52, std = 0.14	0.64, std = 0.21	0.49, std = 0.44	0.52, std = 0.11
η[pN.s/μm]	383, std = 60	96, std = 11	228, std = 39	112, std = 32	519, std = 60	95, std = 15
$\bar{\eta }\;\left[\text{pN}.\text{s}/\mu \text{m}\right]$	274, std = 262	14, std = 86	0.09, std = 194	384, std = 276	0.21, std = 433	119, std = 101
$\alpha \;\left[\text{pN}.\text{s}/\mu {\text{m}}^{3}\right]$	1.94, std = 0.07	2.67, std = 0.08	6.16, std = 0.16	5.0, std = 0.19	3.42, std = 0.20	1.89, std = 0.04
τ[s]	156, std = 84	293, std = 1739	91, std = 47	71, std = 1184	283, std = 1652	135, std = 45
$\sqrt{E\tau /\alpha }\left[\mu \text{m}\right]$	54	28	20	18	53	28

Table of fit parameters.

#### Procedure for analysis of fit uncertainty

In order to estimate the uncertainty in the fit parameters from the 2D analysis, we developed a procedure to analyze the effect of experimental uncertainty on the fit. For each condition, separately, we performed the following analysis:

• We obtained the best fit parameters for the studied condition.
• For each time point of the bead displacement trajectories, we calculated the standard deviation of the mean of experimentally measured displacements. We then averaged over all experimental times to obtain a mean standard deviation of the mean for bead displacement curves, $s_{\text{bd}}$.
• Experimental deformation fields around the bead, measured in a square of side length 80 μm, were then binned in 20 × 20 bins. The standard deviation of the experimental deformation field was then obtained for each bin. We then averaged over all bins which contained more than 3 experimental measurements, to obtain a mean standard deviation for deformation fields, $\sigma_{\text{df}}$.
• A theoretical displacement curve and deformation field was generated from the best fit parameters and evaluated on experimental time points and spatial points.
• Normally distributed random numbers with zero mean and standard deviations $s_{\text{bd}}$ and $\sigma_{\text{df}}$ were added to, respectively, each time point of the theoretical bead displacement curve, and each point of the theoretical deformation field, prior to binning.
• These generated “synthetic” data were then fitted to our theoretical prediction, taking the original fit parameters as an initial guess. For some parameters, we imposed boundaries on the fit parameters returned; namely we imposed τ < 8000 s, λ > 4 · 10^−4^, β > 7.5 · 10^−3^.
• The standard deviation of the resulting fit parameters was then used to generate plots and error bars in Figures 3C and S3C.

## Supplemental Information

Document S1. Figures S1–S4

Document S2. Article plus Supplemental Information

## References

[1] Lecuit T., Lenne P.F., Munro E. (2011). Force generation, transmission, and integration during cell and tissue morphogenesis. Annu. Rev. Cell Dev. Biol..

[2] Heisenberg C.P., Bellaïche Y. (2013). Forces in tissue morphogenesis and patterning. Cell.

[3] Campàs O., Mammoto T., Hasso S., Sperling R.A., O’Connell D., Bischof A.G., Maas R., Weitz D.A., Mahadevan L., Ingber D.E. (2014). Quantifying cell-generated mechanical forces within living embryonic tissues. Nat. Methods.

[4] Bambardekar K., Clément R., Blanc O., Chardès C., Lenne P.F. (2015). Direct laser manipulation reveals the mechanics of cell contacts in vivo. Proc. Natl. Acad. Sci. USA.

[5] Serwane F., Mongera A., Rowghanian P., Kealhofer D.A., Lucio A.A., Hockenbery Z.M., Campàs O. (2017). In vivo quantification of spatially varying mechanical properties in developing tissues. Nat. Methods.

[6] Desprat N., Supatto W., Pouille P.A., Beaurepaire E., Farge E. (2008). Tissue deformation modulates twist expression to determine anterior midgut differentiation in *Drosophila* embryos. Dev. Cell.

[7] Kumar A., Shivashankar G.V. (2012). Mechanical force alters morphogenetic movements and segmental gene expression patterns during *Drosophila* embryogenesis. PLoS ONE.

[8] Doubrovinski K., Swan M., Polyakov O., Wieschaus E.F. (2017). Measurement of cortical elasticity in *Drosophila melanogaster* embryos using ferrofluids. Proc. Natl. Acad. Sci. USA.

[9] Royou A., Field C., Sisson J.C., Sullivan W., Karess R. (2004). Reassessing the role and dynamics of nonmuscle myosin II during furrow formation in early *Drosophila* embryos. Mol. Biol. Cell.

[10] Figard L., Xu H., Garcia H.G., Golding I., Sokac A.M. (2013). The plasma membrane flattens out to fuel cell-surface growth during *Drosophila* cellularization. Dev. Cell.

[11] Lecuit T., Wieschaus E. (2000). Polarized insertion of new membrane from a cytoplasmic reservoir during cleavage of the *Drosophila* embryo. J. Cell Biol..

[12] Lecuit T., Samanta R., Wieschaus E. (2002). slam encodes a developmental regulator of polarized membrane growth during cleavage of the *Drosophila* embryo. Dev. Cell.

[13] Heer N.C., Miller P.W., Chanet S., Stoop N., Dunkel J., Martin A.C. (2017). Actomyosin-based tissue folding requires a multicellular myosin gradient. Development.

[14] Streichan S.J., Lefebvre M.F., Noll N., Wieschaus E.F., Shraiman B.I. (2018). Global morphogenetic flow is accurately predicted by the spatial distribution of myosin motors. eLife.

[15] Etournay R., Popović M., Merkel M., Nandi A., Blasse C., Aigouy B., Brandl H., Myers G., Salbreux G., Jülicher F., Eaton S. (2015). Interplay of cell dynamics and epithelial tension during morphogenesis of the *Drosophila* pupal wing. eLife.

[16] Mayer M., Depken M., Bois J.S., Jülicher F., Grill S.W. (2010). Anisotropies in cortical tension reveal the physical basis of polarizing cortical flows. Nature.

[17] Bonnet I., Marcq P., Bosveld F., Fetler L., Bellaïche Y., Graner F. (2012). Mechanical state, material properties and continuous description of an epithelial tissue. J. R. Soc. Interface.

[18] Harris T.J., Peifer M. (2007). aPKC controls microtubule organization to balance adherens junction symmetry and planar polarity during development. Dev. Cell.

[19] Fabrowski P., Necakov A.S., Mumbauer S., Loeser E., Reversi A., Streichan S., Briggs J.A., De Renzis S. (2013). Tubular endocytosis drives remodelling of the apical surface during epithelial morphogenesis in *Drosophila*. Nat. Commun..

[20] Ray R.P., Matamoro-Vidal A., Ribeiro P.S., Tapon N., Houle D., Salazar-Ciudad I., Thompson B.J. (2015). Patterned anchorage to the apical extracellular matrix defines tissue shape in the developing appendages of *Drosophila*. Dev. Cell.

[21] Diaz-de-la-Loza M.D., Ray R.P., Ganguly P.S., Alt S., Davis J.R., Hoppe A., Tapon N., Salbreux G., Thompson B.J. (2018). Apical and basal matrix remodeling control epithelial morphogenesis. Dev. Cell.

[22] Smutny M., Ákos Z., Grigolon S., Shamipour S., Ruprecht V., Čapek D., Behrndt M., Papusheva E., Tada M., Hof B. (2017). Friction forces position the neural anlage. Nat. Cell Biol..

[23] Harris A.R., Peter L., Bellis J., Baum B., Kabla A.J., Charras G.T. (2012). Characterizing the mechanics of cultured cell monolayers. Proc. Natl. Acad. Sci. USA.

[24] Zhou J., Kim H.Y., Davidson L.A. (2009). Actomyosin stiffens the vertebrate embryo during crucial stages of elongation and neural tube closure. Development.

[25] Mazumdar A., Mazumdar M. (2002). How one becomes many: blastoderm cellularization in *Drosophila melanogaster*. BioEssays.

[26] Foe V.E., Odell G.M., Edgar B.A., Bate M., Martinez Arias A. (1993). Mitosis and morphogenesis in the *Drosophila* embryo: point and counterpoint. The Development of *Drosophila melanogaster*, Volume 1.

[27] Morin X., Daneman R., Zavortink M., Chia W. (2001). A protein trap strategy to detect GFP-tagged proteins expressed from their endogenous loci in *Drosophila*. Proc. Natl. Acad. Sci. USA.

[28] Vasquez C.G., Tworoger M., Martin A.C. (2014). Dynamic myosin phosphorylation regulates contractile pulses and tissue integrity during epithelial morphogenesis. J. Cell Biol..

[29] Aigouy B., Farhadifar R., Staple D.B., Sagner A., Röper J.C., Jülicher F., Eaton S. (2010). Cell flow reorients the axis of planar polarity in the wing epithelium of *Drosophila*. Cell.

[30] Fish M.P., Groth A.C., Calos M.P., Nusse R. (2007). Creating transgenic *Drosophila* by microinjecting the site-specific phiC31 integrase mRNA and a transgene-containing donor plasmid. Nat. Protoc..

[31] Kubala M.H., Kovtun O., Alexandrov K., Collins B.M. (2010). Structural and thermodynamic analysis of the GFP:GFP-nanobody complex. Protein Sci..

[32] Kollmannsberger P., Fabry B. (2007). High-force magnetic tweezers with force feedback for biological applications. Rev. Sci. Instrum..

