# Supplementary material for: In Vivo Force Application Reveals a Fast Tissue Softening and External Friction Increase during Early Embryogenesis
Source: Curr Biol. 2019 May 6;29(9):1564–1571.e6. doi: 10.1016/j.cub.2019.04.010 (PMC6509404; doi:10.1016/j.cub.2019.04.010)
Supplement: Document S1. Figures S1–S4 [file mmc1.pdf]

Current Biology, Volume *29*

Supplemental Information

In Vivo Force Application Reveals a Fast Tissue Softening and External Friction Increase during Early Embryogenesis

Arturo D'Angelo, Kai Dierkes, Carlo Carolis, Guillaume Salbreux, and Jérôme Solon

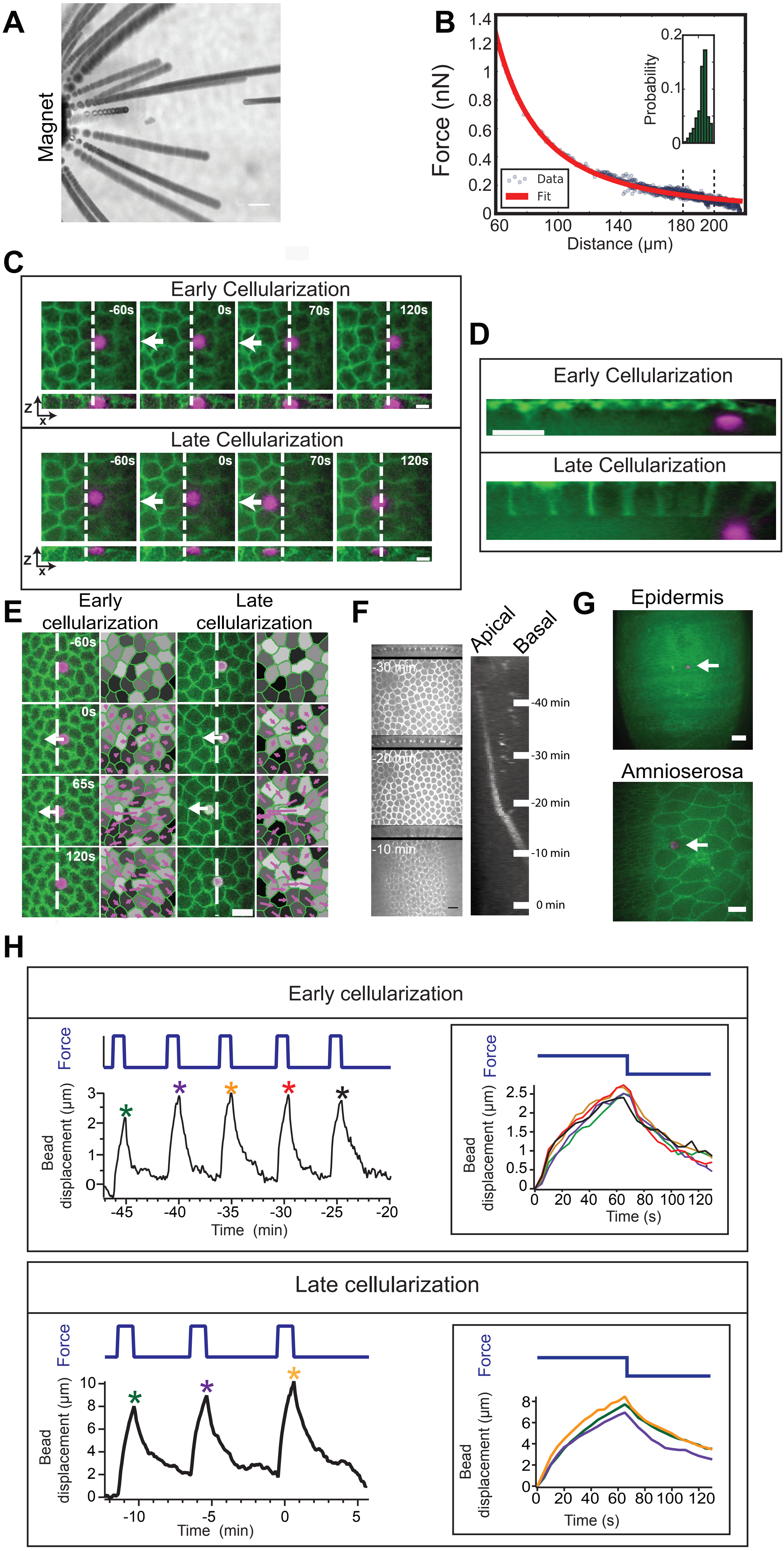

A
Magnet
B
Force (nN)
Distance (μm)
Probability
Data
Fit
C
Early Cellularization
Late Cellularization
-60s
0s
70s
120s
D
Early Cellularization
Late Cellularization
E
Early cellularization
Late cellularization
-60s
0s
65s
120s
F
Apical
Basal
-30 min
-20 min
-10 min
-40 min
0 min
G
Epidermis
Amnioserosa
H
Early cellularization
Late cellularization
Force
Bead displacement (μm)
Time (min)
Time (s)

Figure S1 Force calibration, bead positioning in tissues and ectopic force delivery. Related to Figures 1 and 2 and STAR Methods.

(A) Time projection image of 4.5 μm magnetic beads embedded in PDMS moving toward the magnet during application of the magnetic field generated with a current of 0.3A. The dark lines are bead trajectories. Scale bar: 20 μm

(B) Example of a force-distance calibration curve conducted on 4.5 μm beads (dots) with power law fit (red). The inset shows the probability distribution of bead-magnet distances, between 180 and 200 μm, for the force application experiments used in this work.

(C) Time lapse images showing the kinetics of a bead embedded into a single cell of a Resille-GFP expressing embryo upon force application (~115 pN) and the corresponding reslice along the Z-axis (bottom) at early and late cellularization. The magnetic bead is displaced upon force application in a direction parallel to the apical surface and the vitelline envelope. White arrows indicate the direction of force application and are present in frames in which the force was applied. The white dashed lines indicate the left side of the bead at t=-60s. Scale bar: 5 μm.

(D) Snapshots along the Z-axis of an uncoated $4.5\,\mu m$ bead (magenta) in a Resille-GFP embryo at early (top) and late (bottom) cellularization. The uncoated bead is not located apically and remains in the yolk underneath the blastoderm. Scale bar: 10 μm.

(E) Time lapse sequences showing the kinetics of a bead embedded into a single cell of a Resille-GFP embryo upon force application (~115 pN) at early cellularization (left, t<-16 min relative to gastrulation) and late cellularization (right, t>-16 min relative to gastrulation) and the corresponding velocity fields. Images from the time

lapse are the same than images in Figure 1B showing the corresponding deformation fields. White arrows indicate the direction of force application in frames when the force was applied. White dashed lines indicate the left side of the bead at t=-60s. The velocities are calculated using the centers of mass of individual cells between two consecutive time points. Scale bar: 10 μm.

(F) Snapshots of maximum projected z stacks movie of Sqh-GFP embryo at -30, -20 and -10 min relative to the onset of gastrulation. Right: kymograph of myosin intensity along the z (apico-basal) direction of the tissue, integrated along the A-P axis at the embryo midline, corresponding to the Sqh-GFP embryo shown on the left. A peak of high myosin intensity moves basally during the slow and fast phases of cellularization. The apical side is on the left and the basal side is on the right. The origin of time is set at the onset of gastrulation. Scale bar: 10 μm.

(G) Snapshots of single $4.5\,\mu m$ beads (in magenta) embedded in the ventral epidermis at late embryonic stage (top, scale bar: 20 μm) and in the amnioserosa tissue at dorsal closure stage (bottom, scale bar: 10 μm).

(H) Bead displacement curves during consecutive force applications of ~115 pN: five performed at early cellularization (top) and three at late cellularization (bottom). In these graphs, the origin of time is set to the onset of gastrulation. On the right, the bead displacement curves have been realigned in time using the onset of force application as origin of time. The color code corresponds to that of the asterisks on the graph on the left. At both early and late cellularization, the bead trajectories are close to each other, indicating that the tissue mechanical response does not change following consecutive force applications.

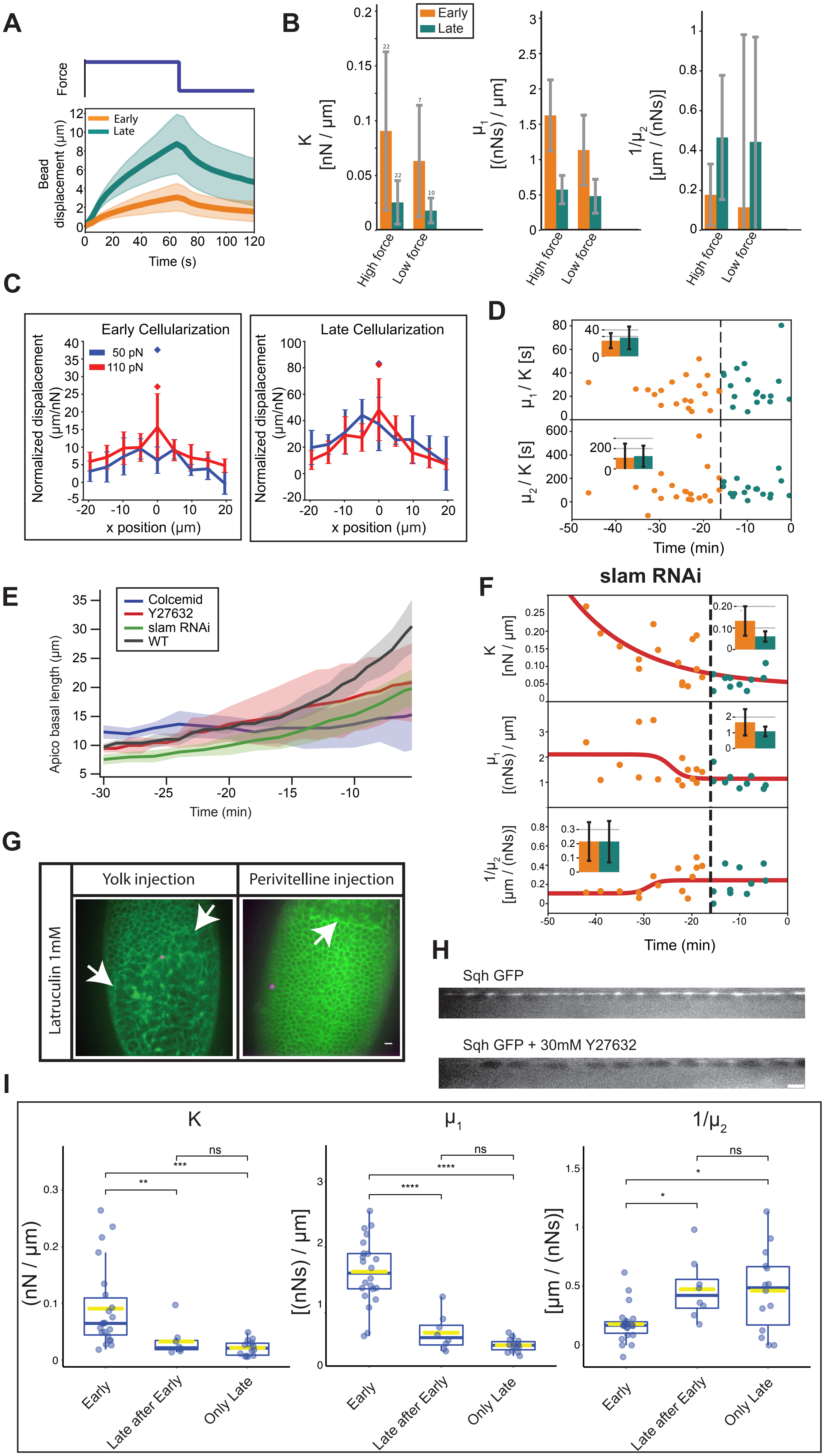
A
Force
Early
Late
Bead displacement (µm)
Time (s)
B
Early
Late
K [nN / µm]
µ1 [(nNs) / µm]
1/µ2 [µm / (nNs)]
High force
Low force
C
Early Cellularization
50 pN
110 pN
Normalized dispalacement (µm/nN)
x position (µm)
Late Cellularization
D
µ1 / K [s]
µ2 / K [s]
Time (min)
E
Colcemid
Y27632
slam RNAi
WT
Apico basal length (µm)
Time (min)
F
slam RNAi
K [nN / µm]
µ1 [(nNs) / µm]
1/µ2 [µm / (nNs)]
Time (min)
G
Latruculin 1mM
Yolk injection
Perivitelline injection
H
Sqh GFP
Sqh GFP + 30mM Y27632
I
K
µ1
1/µ2
(nN / µm)
[(nNs) / µm]
[µm / (nNs)]
ns
***
**
****
*
Early
Late after Early
Only Late

Figure S2 Analysis of bead displacements with a Maxwell Kelvin-Voigt model, kinetics of cellularization and cytoskeletal perturbation. Related to Figure 2.

(A) Average bead displacements for force steps of ~115 pN at early (n=22 applications on 8 embryos) and late (n=22 applications on 11 embryos) cellularization in Resille-GFP embryos. The shaded regions represent standard deviations.

(B) Estimation of stiffness K, viscosity coefficient μ_1 and fluidity $1/\mu_2$ using the Maxwell-Kelvin-Voigt spring-dashpot model for force applications on WT embryos (Resille-GFP) at low force steps (~50pN) and high force steps (~115pN). The three estimated parameters are similar for 50pN and 115pN forces indicating that the tissue behaves linearly in this force regime. Error bars represent standard deviations. ($High_{early}$=22 force applications on 8 embryos, $High_{late}$= 22 on 11 embryos, Low_{early}=7 force applications on 3 embryos, Low_{late}= 10 force applications on 4 embryos).

(C) Average displacements of the tissues in the direction of the applied force, at positions relative to the bead position along the axis of application. Displacements at t=65s after force application are normalized by the pulling force magnitude, for low (~50 pN) and high (~115 pN) force amplitudes at early (left) and late (right) cellularization. The rhombuses indicate maximal bead displacements normalized by the force magnitude, and error bars show standard deviations. Normalized displacements at ~50pN and ~115pN at early and late cellularization are close to each other, indicative of a linear response of the tissue in this regime. ($High_{early}$=22 force applications on 8 embryos, $High_{late}$= 18 force applications on 10 embryos, Low_{early}=4 force applications on 2 embryos, Low_{late}= 5 force applications on 2 embryos).

(D) Viscoelastic timescales ${}^{\mu_1}/_{K}$ and ${}^{\mu_2}/_{K}$ as a function of time. The origin of time is defined as the onset of gastrulation. Dashed lines indicate the separation between early and late stages of cellularization. Insets show the average timescales for each stage and error bars indicate standard deviations. Five outlier points with extreme values ($\sim 10^9 s$) have been excluded from the graph.

(E) Measure of the apico-basal length over time using Resille GFP in WT embryos (n=3 embryos), Y27632 (n=3 embryos), Colcemid (n=3 embryos) and slam RNAi (n=7 embryos) treated embryos during cellularization. The origin of time is set at the onset of gastrulation. The shaded regions indicate standard deviations.

(F) Effective parameters K, μ_1 and μ_2 as a function of developmental time relative to the onset of gastrulation in slam RNAi embryos. Each dot represents a single force application of ~115 pN. The red lines are fits to the experimental data performed with a sigmoid function (see STAR Methods). The black dashed lines show the average of the time in the middle of the step from the sigmoid fits performed on WT embryos, and are used to distinguish between force applications in the early (orange) and late (blue) phases of cellularization. The insets show the average parameters in the early and late phases. The error bars indicate standard deviations. In this situation, the shift is less pronounced and the sigmoid fit fails to identify a clear shift in mechanical parameters. In the case of the parameter K, the sigmoid fitting procedure did not yield a step-like change in mechanics between early and late cellularization. ($slam_{early}$=15 force applications on 5 embryos, $slam_{late}$ =10 force applications on 4 embryos).

(G) Snapshots of Resille embryos injected with Latruculin A in the yolk (left) and in the perivetelline space (right). In both cases, Latruculin A injection generates a loss

of tissue integrity in a region of the blastoderm (indicated by the white arrows) while the rest of blastoderm is unaffected. Because the region of the blastoderm affected by the drug completely loses its integrity, we did not perform mechanical probing under Latrunculin A conditions. Scale bar: 10 μm.

(H) Snapshots showing Sqh-GFP embryos at early cellularization along the Z-axis, both uninjected (Top) and injected with Y27632 (Bottom). In control embryos, myosin accumulates around the nuclei (Top), whilst In Y27632 injected embryos myosin localization around the nucleus is not present. Scale bar: 5 μm

(I) Effective parameters K, μ_1 and $1/\mu_2$, estimated from force pulling experiments performed in embryos at early cellularization (labeled Early), at late cellularization in embryos that had been pulled at both early and late cellularization (Late after Early) and at late cellularization in embryos only pulled at late cellularization (labeled Only Late). There are no significant differences in the effective parameters between Late after Early and Only Late application, ruling out the possibility that repeated force applications influence the observed changes in mechanics between early and late cellularization. The box plot encloses 50% of the data around the median, yellow lines represent the mean of the data. The upper/lower whisker extends from the hinge to the largest/smallest value no further than 1.5 * IQR from the hinge respectively (where IQR is the inter-quartile range, or distance between the first and third quartiles). Each dot is a single force application. (Early = 22 force applications on 8 embryos, Late after Early = 8 force applications on 5 embryos and Only Late = 14 force applications on 6 embryos). NS= not significant, *P<0.05, **P<0.01, ***P<0.001, ****P<0.0001.

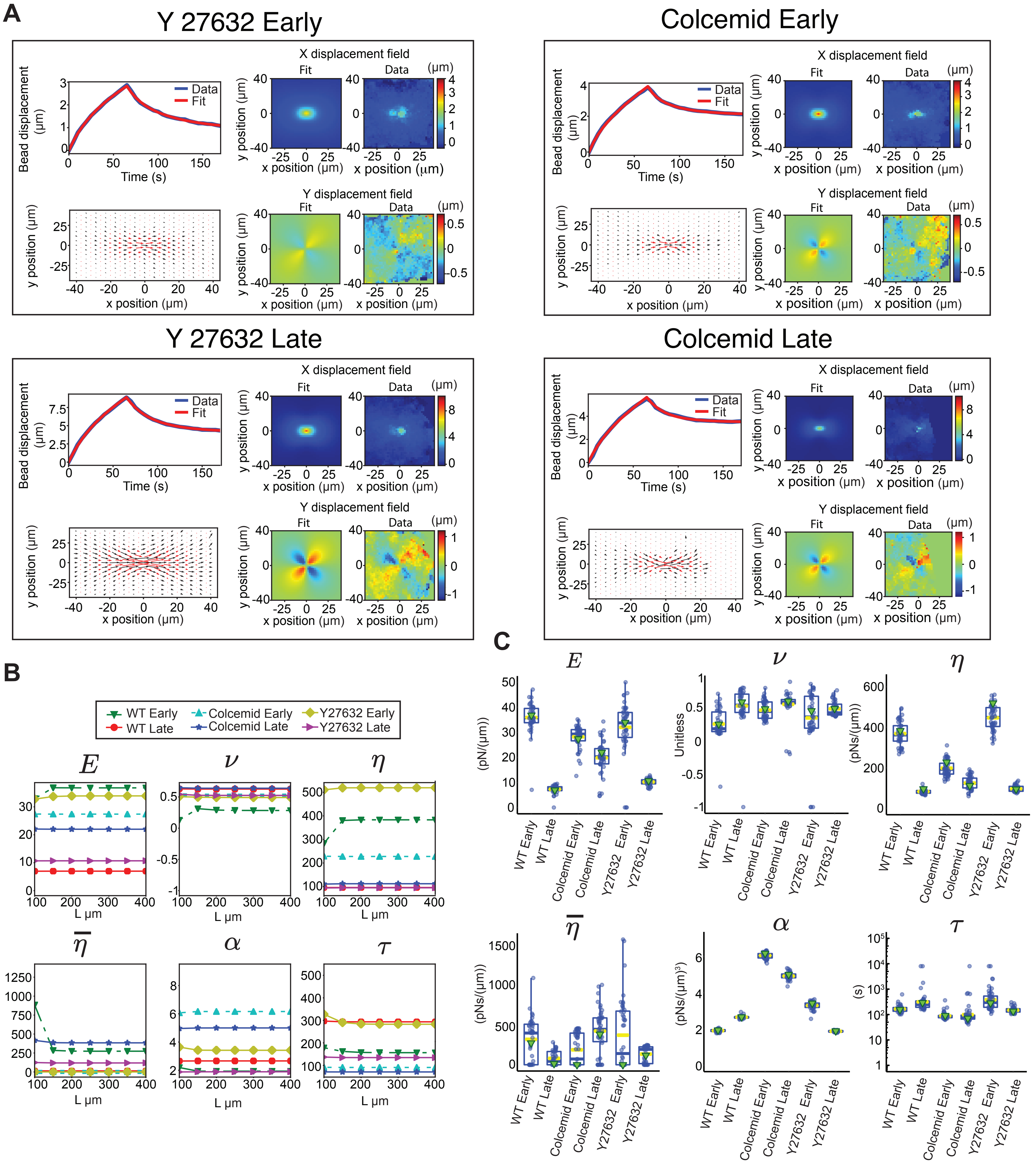
A
Y 27632 Early
Colcemid Early
Y 27632 Late
Colcemid Late
X displacement field
Y displacement field
Fit
Data
(μm)
Bead displacement (μm)
Time (s)
Data
Fit
x position (μm)
y position (μm)
B
WT Early
WT Late
Colcemid Early
Colcemid Late
Y27632 Early
Y27632 Late
L μm
C
Unitless
(pN/(μm))
(pNs/(μm))
(pNs/(μm)^3)
(s)
WT Early
WT Late
Colcemid Early
Colcemid Late
Y27632 Early
Y27632 Late

Figure S3 Analysis of bead displacements and deformation fields, using a 2D continuum mechanics model. Related to Figure 3 and STAR Methods.

(A) Average bead displacements and deformation fields at early and late cellularization in Y27632 and Colcemid treated embryos, with respective fits of both the bead displacements and deformation fields using a 2D continuum mechanics model. For all conditions, the average experimental and fitted bead displacements are shown in the top-left panel, the experimental and fitted deformation fields in the bottom-left panel (black and red arrows, respectively), and fitted and experimental x- and y-deformations in the middle and right panels, top and bottom. $Y27632_{Early}$= 24 force applications on 7 embryos for the bead displacement and 17 force applications on 5 embryos for the deformation field. $Y27632_{Late}$= 22 force applications on 7 embryos for the bead displacement and 13 force applications on 5 embryos for the deformation field. $Colcemid_{Early}$= 19 force applications on 5 embryos for the bead displacement and 13 force application on 5 embryos for the deformation field, $Colcemid_{Late}$= 9 force applications on 3 embryos for the bead displacement and 5 force applications on 2 embryos for the deformation field.

(B) Mechanical parameters extracted from the fit of the bead displacement and deformation field as a function of the size L of the theoretical 2D viscoelastic tissue in the cases of WT, Colcemid and Y 27632 at early and late cellularization. Beyond 200μm, parameters are largely independent on the size of the 2D tissue.

(C) Mechanical parameters extracted from the fit of experimental data using our continuum description. Values of the 2D elastic Young's modulus E, the 2D poisson ratio ν, the shear viscosity η, the bulk viscosity $\bar{\eta}$, the friction coefficient α, and the Maxwell viscoelastic timescale τ are shown for WT, Colcemid and Y27632 at early

and late cellularization. Each blue dot is a single fit value arising from the fit uncertainty analysis (STAR Methods). The box plot encloses 50% of the data around the median, the yellow lines represent the mean of the data and the green triangle shows the value obtained from the fit of the data (Figure 3B for the WT and Figure S3A for Colcemid and Y27632). The upper/lower whisker extends from the hinge to the largest/smallest value no further than 1.5 * IQR from the hinge respectively (where IQR is the inter-quartile range, or distance between the first and third quartiles).

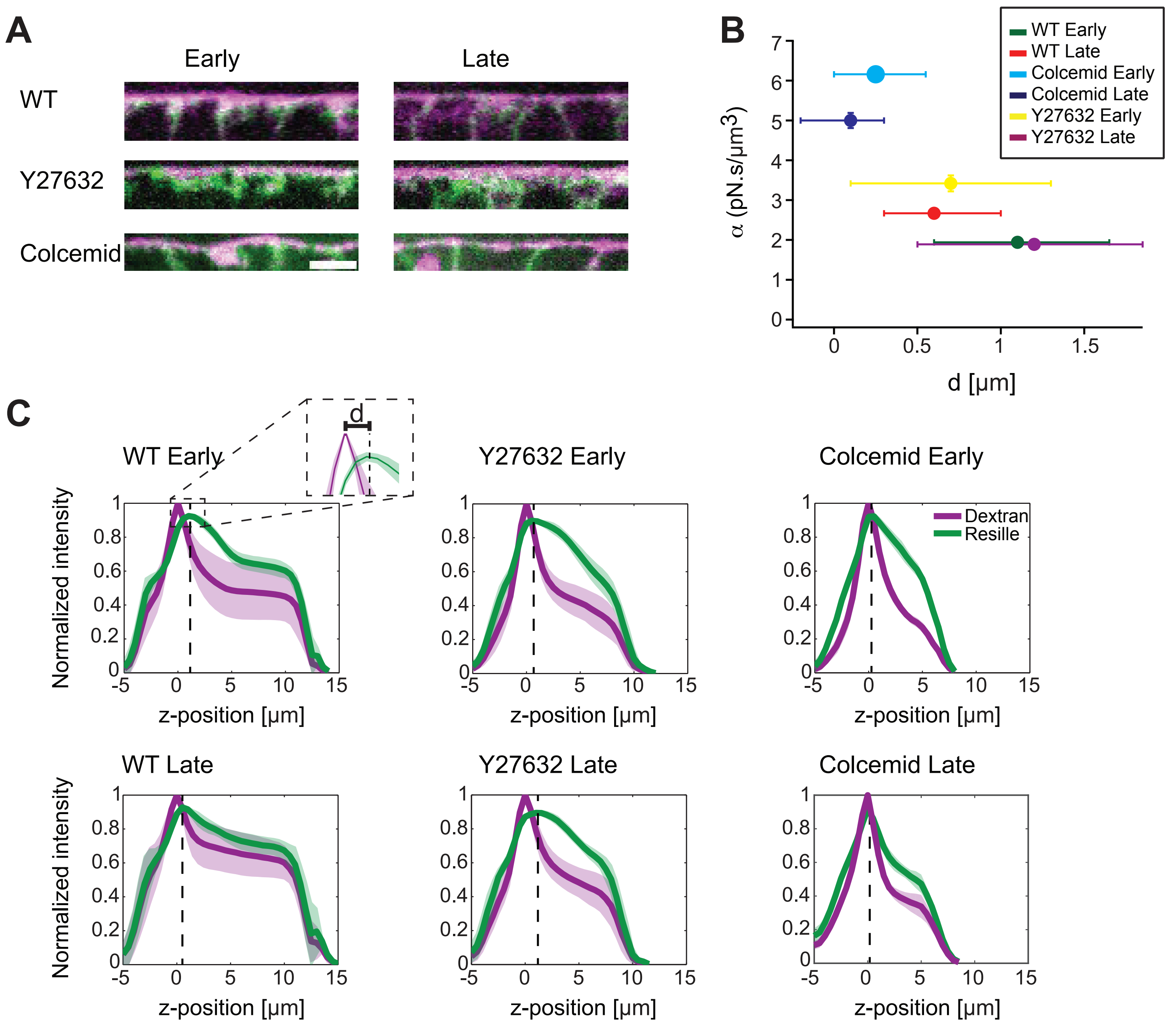

A
Early
Late
WT
Y27632
Colcemid
B
WT Early
WT Late
Colcemid Early
Colcemid Late
Y27632 Early
Y27632 Late
α (pN.s/μm3)
d [μm]
C
d
WT Early
Y27632 Early
Colcemid Early
Dextran
Resille
WT Late
Y27632 Late
Colcemid Late
Normalized intensity
z-position [μm]

Figure S4 Estimation of perivitelline space width for WT, Y27632 and Colcemid treated embryos. Related to Figure 4.

(A) Z-reslices along the A-P axis of the embryo showing dextran texas red injected in the perivitelline space (in magenta) and Resille GFP signal (in green) for WT, Y27632, and Colcemid injected embryos, at early and late cellularization. Scale bar: 5μm.

(B) The graph shows the friction coefficient α (see Figure 3C and Figure S3C) as a function of the distance between the peak intensities of dextran and Resille GFP (used as a proxy for the perivitelline space width), as measured in C, for WT, Y27632, and Colcemid injected embryos. We observe an inverse relationship between the friction coefficient and the inter-peak distance. The x-error bars represent the width of the Resille peak at 99% of the maximal height and the y-error bars represent the standard deviation of the friction coefficient obtained from the noise analysis (see STAR Methods).

(C) Average fluorescence intensity profiles of the dextran and cellular membrane marker Resille-GFP for WT, Y27632, and Colcemid treated embryos, at early and late cellularization (WT_{early}=22 profiles on 3 embryos, WT_{late}=21 profiles on 3 embryos, $Y27632_{early}$=26 profiles on 3 embryos, $Y27632_{late}$=30 profiles on 3 embryos, $Colc_{early}$=10 profiles on 2 embryos, $Colc_{late}$=10 profiles on 2 embryos). The dashed line shows the position of the maximum fluorescence peak for Resille (see STAR Methods) and the origin of z-position is set by the peak of dextran fluorescence. Error bars show standard deviation. The inset highlights the shift between the Resille and dextran peaks used as a proxy for the perivitelline space width.
